# Supplementary material for: Insight into the evolution of microbial metabolism from the deep-branching bacterium, Thermovibrio ammonificans
Source: eLife. 2017 Apr 24;6:e18990. doi: 10.7554/eLife.18990 (PMC5441870; doi:10.7554/eLife.18990)
Supplement: Figure 3—source data 1. — DOI: http://dx.doi.org/10.7554/eLife.18990.008 [file elife-18990-fig3-data1.docx]

**Figure 3-sourcet data 1.** List of the proteins identified in the proteome of *T. ammonificans* grown under nitrate reducing conditions.

| **Locus** | **Product name** | ***Gene Name*** | **Accession number** | **MW (kDa)** | **NSAF %** |
| --- | --- | --- | --- | --- | --- |
| Theam_0001 | anthranilate synthase component I | *trpE* | YP_004150616.1 | 55 | **0.09** |
| Theam_0005 | glycosyl transferase group 1 | *alg1* | YP_004150620.1 | 64 | **0.09** |
| Theam_0007 | DNA polymerase III, beta subunit | *dnaN* | YP_004150622.1 | 39 | **0.25** |
| Theam_0010 | Polyprenyl synthetase | *ggpps* | YP_004150625.1 | 32 | **0.10** |
| Theam_0011 | deoxyxylulose-5-phosphate synthase | *dxs* | YP_004150626.1 | 68 | **0.02** |
| Theam_0025 | phage protein |  | YP_004150640.1 | 40 | **0.04** |
| Theam_0045 | hypothetical protein |  | YP_004150660.1 | 9 | **0.02** |
| Theam_0050 | hypothetical protein |  | YP_004150665.1 | 35 | **0.03** |
| Theam_0051 | hypothetical protein |  | YP_004150666.1 | 33 | **0.01** |
| Theam_0069 | Rhodanese domain protein |  | YP_004150684.1 | 14 | **1.06** |
| Theam_0070 | regulatory protein ArsR | *arsR* | YP_004150685.1 | 14 | **0.04** |
| Theam_0071 | carbonic anhydrase | *cah* | YP_004150686.1 | 25 | **0.04** |
| Theam_0073 | methylthioadenosine phosphorylase | *mtaP* | YP_004150688.1 | 32 | **0.58** |
| Theam_0074 | 50S ribosomal Protein L19 (hypothetical protein) | *rpL19* | YP_004150689.1 | 11 | **0.09** |
| Theam_0075 | imidazoleglycerol phosphate synthase, cyclase subunit | *hisF* | YP_004150690.1 | 27 | **0.06** |
| Theam_0076 | thiamine biosynthesis protein ThiC | *thiC* | YP_004150691.1 | 48 | **0.23** |
| Theam_0077 | thiazole biosynthesis enzyme | *thi1* | YP_004150692.1 | 29 | **1.13** |
| Theam_0078 | hypothetical protein |  | YP_004150693.1 | 48 | **0.06** |
| Theam_0079 | amidohydrolase | *ah* | YP_004150694.1 | 45 | **0.02** |
| Theam_0080 | orotate phosphoribosyltransferase | *pyrE* | YP_004150695.1 | 22 | **0.10** |
| Theam_0081 | Polyprenyl synthetase | *idsB* | YP_004150696.1 | 36 | **0.11** |
| Theam_0084 | NAD-dependent epimerase/dehydratase |  | YP_004150699.1 | 37 | **0.05** |
| Theam_0085 | chromosome segregation protein SMC | *smc* | YP_004150700.1 | 134 | **0.01** |
| Theam_0088 | ribosome recycling factor | *frr* | YP_004150703.1 | 21 | **0.03** |
| Theam_0089 | uridylate kinase | *pyrH* | YP_004150704.1 | 26 | **0.19** |
| Theam_0090 | translation elongation factor Ts | *tsf* | YP_004150705.1 | 22 | **0.46** |
| Theam_0091 | ribosomal protein S2 | *rpsB_bact* | YP_004150706.1 | 33 | **0.46** |
| Theam_0092 | 5-oxoprolinase (ATP-hydrolyzing) | *opla* | YP_004150707.1 | 56 | **0.03** |
| Theam_0093 | 5-oxoprolinase (ATP-hydrolyzing) | *opla* | YP_004150708.1 | 71 | **0.11** |
| Theam_0094 | ubiquinone/menaquinone biosynthesis methyltransferase | *men_ubi* | YP_004150709.1 | 24 | **0.01** |
| Theam_0095 | hypothetical protein |  | YP_004150710.1 | 44 | **0.00** |
| Theam_0098 | apurinic endonuclease Apn1 | *nfo* | YP_004150713.1 | 32 | **0.02** |
| Theam_0100 | hypothetical protein |  | YP_004150715.1 | 13 | **0.10** |
| Theam_0101 | hypothetical protein |  | YP_004150716.1 | 15 | **0.02** |
| Theam_0102 | hypothetical protein |  | YP_004150717.1 | 22 | **0.02** |
| Theam_0103 | hypothetical protein |  | YP_004150718.1 | 12 | **0.05** |
| Theam_0104 | beta-lactamase domain protein |  | YP_004150719.1 | 32 | **0.09** |
| Theam_0105 | Roadblock/LC7 family protein |  | YP_004150720.1 | 13 | **0.01** |
| Theam_0106 | pyrimidine-nucleoside phosphorylase | *pynp* | YP_004150721.1 | 46 | **0.05** |
| Theam_0108 | AMMECR1 domain protein |  | YP_004150723.1 | 22 | **0.03** |
| Theam_0122 | outer membrane assembly lipoprotein YfiO | *yfiO* | YP_004150737.1 | 37 | **0.01** |
| Theam_0126 | protein of unknown function DUF481 |  | YP_004150741.1 | 25 | **0.03** |
| Theam_0129 | histone family protein DNA-binding protein |  | YP_004150744.1 | 11 | **0.13** |
| Theam_0132 | non-canonical NTP pyrophosphatase rdgB/HAM1 family | *rdgB* | YP_004150747.1 | 22 | **0.12** |
| Theam_0133 | ribonuclease PH | *RNasePH* | YP_004150748.1 | 26 | **0.06** |
| Theam_0136 | ATP-dependent Clp protease, proteolytic subunit ClpP | *clpP* | YP_004150751.1 | 22 | **0.31** |
| Theam_0137 | 3,4-dihydroxy-2-butanone 4-phosphate synthase | *ribA* | YP_004150752.1 | 48 | **0.03** |
| Theam_0139 | diguanylate phosphodiesterase CDS |  | YP_004150753.1 | 77 | **0.00** |
| Theam_0140 | shikimate 5-dehydrogenase | *aroE* | YP_004150754.1 | 30 | **0.05** |
| Theam_0142 | ribosomal protein L27 | *rpl27* | YP_004150756.1 | 9 | **0.17** |
| Theam_0143 | ribosomal protein L21 | *rpl21* | YP_004150757.1 | 11 | **0.46** |
| Theam_0145 | inosine-5'-monophosphate dehydrogenase | *impdh* | YP_004150759.1 | 53 | **0.53** |
| Theam_0146 | argininosuccinate lyase | *argH* | YP_004150760.1 | 52 | **0.15** |
| Theam_0147 | Transketolase domain-containing protein |  | YP_004150761.1 | 32 | **0.11** |
| Theam_0148 | Transketolase central region |  | YP_004150762.1 | 34 | **0.36** |
| Theam_0149 | carboxyl-terminal protease | *prc* | YP_004150763.1 | 49 | **0.02** |
| Theam_0151 | hypothetical protein |  | YP_004150765.1 | 37 | **0.02** |
| Theam_0155 | Radical SAM domain protein |  | YP_004150769.1 | 40 | **0.00** |
| Theam_0157 | chemotaxis sensory transducer |  | YP_004150771.1 | 68 | **0.06** |
| Theam_0159 | dihydroorotate dehydrogenase family protein | *pyrD1* | YP_004150773.1 | 32 | **0.11** |
| Theam_0161 | Tetratricopeptide TPR_1 repeat-containing protein |  | YP_004150775.1 | 67 | **0.01** |
| Theam_0163 | peptidase M17 leucyl aminopeptidase domain protein |  | YP_004150777.1 | 52 | **0.03** |
| Theam_0165 | chemotaxis sensory transducer |  | YP_004150779.1 | 86 | **0.02** |
| Theam_0168 | phosphoribosylformimino-5-aminoimidazole carboxamide ribotide isomerase | *hisA* | YP_004150782.1 | 27 | **0.15** |
| Theam_0170 | quinolinate synthetase complex, A subunit | *nadA* | YP_004150784.1 | 35 | **0.01** |
| Theam_0171 | GMP synthase, large subunit | *guaA* | YP_004150785.1 | 59 | **0.12** |
| Theam_0179 | adenylosuccinate synthetase | *purA* | YP_004150793.1 | 48 | **0.09** |
| Theam_0180 | histidyl-tRNA synthetase 2 | *hisS2* | YP_004150794.1 | 48 | **0.01** |
| Theam_0181 | threonine synthase | *thrC* | YP_004150795.1 | 38 | **0.16** |
| Theam_0182 | dihydrodipicolinate synthase | *dapA* | YP_004150796.1 | 32 | **0.35** |
| Theam_0183 | hypothetical protein |  | YP_004150797.1 | 40 | **0.00** |
| Theam_0184 | dihydrodipicolinate reductase | *dapB* | YP_004150798.1 | 28 | **0.26** |
| Theam_0188 | hypothetical protein |  | YP_004150802.1 | 101 | **0.01** |
| Theam_0190 | homoserine dehydrogenase | *ak1h* | YP_004150804.1 | 47 | **0.09** |
| Theam_0191 | aminotransferase class I and II |  | YP_004150805.1 | 46 | **0.09** |
| Theam_0195 | TonB-dependent receptor plug |  | YP_004150809.1 | 67 | **0.08** |
| Theam_0197 | Citryl-CoA lyase | *citE* | YP_004150811.1 | 37 | **0.06** |
| Theam_0198 | LysR substrate-binding | *lysR* | YP_004150812.1 | 34 | **0.01** |
| Theam_0201 | 3-isopropylmalate dehydratase, large subunit | *haco* | YP_004150815.1 | 46 | **0.13** |
| Theam_0205 | nicotinate (nicotinamide) nucleotide adenylyltransferase |  | YP_004150819.1 | 25 | **0.03** |
| Theam_0206 | Phosphoglycerate kinase | *pgk* | YP_004150820.1 | 45 | **0.17** |
| Theam_0210 | Peptidoglycan-binding lysin domain |  | YP_004150824.1 | 45 | **0.00** |
| Theam_0215 | methionyl-tRNA formyltransferase | *fmt* | YP_004150829.1 | 35 | **0.02** |
| Theam_0217 | protein of unknown function DUF89 |  | YP_004150831.1 | 34 | **0.03** |
| Theam_0218 | phosphoribosylaminoimidazole carboxylase, catalytic subunit | *purE* | YP_004150832.1 | 17 | **0.21** |
| Theam_0220 | translation elongation factor P | *efp* | YP_004150834.1 | 21 | **0.06** |
| Theam_0221 | acetyl-CoA carboxylase, biotin carboxyl carrier protein | *accB* | YP_004150835.1 | 16 | **0.23** |
| Theam_0222 | acetyl-CoA carboxylase, biotin carboxylase | *accC* | YP_004150836.1 | 49 | **0.03** |
| Theam_0223 | Like-Sm ribonucleoprotein core |  | YP_004150837.1 | 17 | **0.04** |
| Theam_0227 | aminotransferase class V - serine glyoxylate | *sgt* | YP_004150841.1 | 42 | **0.17** |
| Theam_0228 | peptidylprolyl isomerase FKBP-type |  | YP_004150842.1 | 16 | **0.05** |
| Theam_0229 | flavin reductase domain protein FMN-binding |  | YP_004150843.1 | 21 | **0.11** |
| Theam_0230 | UbiD family decarboxylase | *ubid* | YP_004150844.1 | 56 | **0.00** |
| Theam_0231 | NIF3 containing protein of unknown function DUF34 |  | YP_004150845.1 | 28 | **0.04** |
| Theam_0233 | ATP-dependent protease La | *lon* | YP_004150847.1 | 92 | **0.03** |
| Theam_0234 | UTP-glucose-1-phosphate uridylyltransferase | *galU* | YP_004150848.1 | 34 | **0.12** |
| Theam_0235 | protein of unknown function Met10 |  | YP_004150849.1 | 45 | **0.02** |
| Theam_0237 | carbamoyl-phosphate synthase, small subunit | *cpsS* | YP_004150851.1 | 42 | **0.08** |
| Theam_0238 | periplasmic solute binding protein |  | YP_004150852.1 | 32 | **0.03** |
| Theam_0239 | glutamine amidotransferase of anthranilate synthase | *trpG_papA* | YP_004150853.1 | 21 | **0.02** |
| Theam_0244 | dihydrouridine synthase DuS | *dus* | YP_004150858.1 | 35 | **0.03** |
| Theam_0245 | ribosomal protein S15 | *S15* | YP_004150859.1 | 11 | **0.09** |
| Theam_0246 | polyribonucleotide nucleotidyltransferase | *pnp* | YP_004150860.1 | 79 | **0.19** |
| Theam_0247 | deoxyUTP pyrophosphatase | *dut* | YP_004150861.1 | 32 | **0.06** |
| Theam_0250 | 2-isopropylmalate synthase | *leuA* | YP_004150864.1 | 56 | **0.20** |
| Theam_0252 | ribosomal protein S12 | *rpsL* | YP_004150866.1 | 14 | **0.27** |
| Theam_0253 | ribosomal protein S7 | *rpsG* | YP_004150867.1 | 18 | **0.07** |
| Theam_0254 | translation elongation factor G | *efG* | YP_004150868.1 | 78 | **0.88** |
| Theam_0256 | ribosomal protein S10 | *rpsJ* | YP_004150870.1 | 12 | **0.96** |
| Theam_0257 | 50S ribosomal protein L3 | *rpl3* | YP_004150871.1 | 22 | **0.34** |
| Theam_0258 | ribosomal protein L4/L1e | *rpsE* | YP_004150872.1 | 24 | **0.14** |
| Theam_0259 | Ribosomal protein L25/L23 | *rplL* | YP_004150873.1 | 12 | **0.44** |
| Theam_0260 | ribosomal protein L2 | *rplB* | YP_004150874.1 | 30 | **0.26** |
| Theam_0261 | ribosomal protein S19 | *rpsS* | YP_004150875.1 | 11 | **0.08** |
| Theam_0262 | ribosomal protein L22 | *rplV* | YP_004150876.1 | 15 | **0.38** |
| Theam_0263 | ribosomal protein S3 | *rpsC* | YP_004150877.1 | 27 | **0.29** |
| Theam_0264 | ribosomal protein L16 | *rplP* | YP_004150878.1 | 16 | **0.36** |
| Theam_0266 | 30S ribosomal protein S17 | *rps17* | YP_004150880.1 | 12 | **0.18** |
| Theam_0267 | ribosomal protein L14 | *rplN* | YP_004150881.1 | 13 | **1.18** |
| Theam_0268 | ribosomal protein L24 | *rplX* | YP_004150882.1 | 13 | **0.13** |
| Theam_0269 | 50S ribosomal protein L5 | *rpl5* | YP_004150883.1 | 21 | **0.56** |
| Theam_0271 | ribosomal protein S8 | *rps8* | YP_004150885.1 | 16 | **0.44** |
| Theam_0272 | ribosomal protein L6 | *rpl6* | YP_004150886.1 | 20 | **0.23** |
| Theam_0273 | ribosomal protein L18 | *rpl18* | YP_004150887.1 | 14 | **0.39** |
| Theam_0274 | ribosomal protein S5 | *rpsE* | YP_004150888.1 | 20 | **0.34** |
| Theam_0275 | ribosomal protein L30 | *rpmD* | YP_004150889.1 | 7 | **0.19** |
| Theam_0276 | ribosomal protein L15 | *rplO* | YP_004150890.1 | 17 | **0.04** |
| Theam_0278 | adenylate kinase | *adk* | YP_004150892.1 | 21 | **0.27** |
| Theam_0279 | methionine aminopeptidase, type I | *map1* | YP_004150893.1 | 28 | **0.02** |
| Theam_0282 | 30S ribosomal protein S13 | *rps13* | YP_004150896.1 | 14 | **0.37** |
| Theam_0283 | 30S ribosomal protein S11 | *rps11* | YP_004150897.1 | 14 | **0.16** |
| Theam_0284 | ribosomal protein S4 | *rpsD* | YP_004150898.1 | 25 | **0.24** |
| Theam_0285 | DNA-directed RNA polymerase, alpha subunit | *rpoA* | YP_004150899.1 | 35 | **0.22** |
| Theam_0286 | ribosomal protein L17 | *rpl17* | YP_004150900.1 | 15 | **0.24** |
| Theam_0288 | septum site-determining protein MinD | *minD* | YP_004150902.1 | 29 | **0.01** |
| Theam_0291 | spermidine synthase | *speE* | YP_004150905.1 | 33 | **0.14** |
| Theam_0292 | protein of unknown function DUF43 |  | YP_004150906.1 | 40 | **0.30** |
| Theam_0295 | purine or other phosphorylase family 1 |  | YP_004150909.1 | 33 | **0.00** |
| Theam_0297 | ribosomal 5S rRNA E-loop binding protein Ctc/L25/TL5 | *ctc* | YP_004150911.1 | 22 | **0.10** |
| Theam_0298 | peptidyl-tRNA hydrolase | *pth* | YP_004150912.1 | 21 | **0.03** |
| Theam_0299 | ribosomal protein S6 | *S6* | YP_004150913.1 | 15 | **0.34** |
| Theam_0301 | ribosomal protein S18 | *S18* | YP_004150915.1 | 10 | **0.02** |
| Theam_0303 | ribosomal protein L9 | *rpl9* | YP_004150917.1 | 17 | **0.13** |
| Theam_0304 | S-adenosylmethionine decarboxylase related | *speD* | YP_004150918.1 | 20 | **0.21** |
| Theam_0306 | endoribonuclease L-PSP | *lpsp* | YP_004150920.1 | 14 | **0.06** |
| Theam_0307 | regulatory protein ArsR | *arsR* | YP_004150921.1 | 12 | **0.06** |
| Theam_0308 | acetyl-CoA carboxylase, carboxyl transferase, beta subunit | *accD* | YP_004150922.1 | 31 | **0.01** |
| Theam_0310 | transcription-repair coupling factor | *mfd* | YP_004150924.1 | 119 | **0.00** |
| Theam_0311 | Ankyrin |  | YP_004150925.1 | 26 | **0.01** |
| Theam_0313 | hypothetical protein |  | YP_004150927.1 | 43 | **0.01** |
| Theam_0315 | excinuclease ABC, A subunit | *uvra* | YP_004150929.1 | 105 | **0.00** |
| Theam_0316 | DsrE family protein |  | YP_004150930.1 | 13 | **0.24** |
| Theam_0324 | CMP/dCMP deaminase zinc-binding |  | YP_004150938.1 | 14 | **0.07** |
| Theam_0325 | response regulator receiver |  | YP_004150939.1 | 26 | **0.03** |
| Theam_0326 | hypothetical protein |  | YP_004150940.1 | 22 | **0.06** |
| Theam_0327 | Glutamate synthase (ferredoxin) | *gltA* | YP_004150941.1 | 159 | **0.17** |
| Theam_0328 | phosphoribosylaminoimidazolecarboxamide formyltransferase/IMP cyclohydrolase | *purH* | YP_004150942.1 | 59 | **0.14** |
| Theam_0331 | alanine racemase | *alr* | YP_004150945.1 | 39 | **0.01** |
| Theam_0332 | ribosomal L11 methyltransferase | *rpl11* | YP_004150946.1 | 30 | **0.01** |
| Theam_0333 | PHP domain protein |  | YP_004150947.1 | 31 | **0.01** |
| Theam_0334 | Protein of unknown function DUF2081 |  | YP_004150948.1 | 74 | **0.00** |
| Theam_0337 | heat shock protein HslVU, ATPase subunit HslU | *hslU* | YP_004150951.1 | 55 | **0.01** |
| Theam_0338 | peptidase M24 | *pM24* | YP_004150952.1 | 38 | **0.03** |
| Theam_0342 | CRISPR-associated protein, Csx11 family |  | YP_004150956.1 | 123 | **0.00** |
| Theam_0344 | CRISPR-associated RAMP protein, Cmr1 family |  | YP_004150958.1 | 50 | **0.00** |
| Theam_0347 | 3-isopropylmalate dehydratase, small subunit | *leuA* | YP_004150961.1 | 18 | **0.19** |
| Theam_0348 | 3-isopropylmalate dehydrogenase | *leuB* | YP_004150962.1 | 40 | **0.38** |
| Theam_0349 | aspartate-semialdehyde dehydrogenase | *dhaS* | YP_004150963.1 | 37 | **0.23** |
| Theam_0350 | hypothetical protein |  | YP_004150964.1 | 14 | **0.02** |
| Theam_0351 | metal-dependent hydrolase |  | YP_004150965.1 | 25 | **0.18** |
| Theam_0354 | Peptidase M23 | *pM23* | YP_004150968.1 | 39 | **0.00** |
| Theam_0356 | TrkA-N domain protein |  | YP_004150970.1 | 48 | **0.03** |
| Theam_0357 | cytochrome c-type biogenesis protein CcsB | *ccsB* | YP_004150971.1 | 38 | **0.01** |
| Theam_0358 | ResB family protein |  | YP_004150972.1 | 50 | **0.00** |
| Theam_0360 | metal dependent phophohydrolase | *hdig* | YP_004150974.1 | 55 | **0.01** |
| Theam_0362 | ATPase AAA-2 domain protein |  | YP_004150976.1 | 94 | **0.01** |
| Theam_0363 | protein of unknown function DUF558 |  | YP_004150977.1 | 26 | **0.07** |
| Theam_0365 | Appr-1-p processing domain protein |  | YP_004150979.1 | 19 | **0.12** |
| Theam_0373 | 2-isopropylmalate synthase/homocitrate synthase family protein | *leu1* | YP_004150987.1 | 60 | **0.16** |
| Theam_0374 | aspartate kinase | *aspK* | YP_004150988.1 | 44 | **0.40** |
| Theam_0375 | molybdenum cofactor synthesis domain protein | *moeA* | YP_004150989.1 | 44 | **0.00** |
| Theam_0376 | glyceraldehyde-3-phosphate dehydrogenase | *gapor* | YP_004150990.1 | 75 | **0.01** |
| Theam_0377 | N-acetyl-gamma-glutamyl-phosphate reductase | *argC* | YP_004150991.1 | 39 | **0.09** |
| Theam_0378 | ribosomal protein S9 | *rps9* | YP_004150992.1 | 15 | **0.44** |
| Theam_0379 | ribosomal protein L13 | *rplM* | YP_004150993.1 | 16 | **0.02** |
| Theam_0385 | ribulose-phosphate 3-epimerase | *rpe* | YP_004150999.1 | 24 | **0.13** |
| Theam_0386 | ribose-phosphate pyrophosphokinase | *rpppk* | YP_004151000.1 | 34 | **0.73** |
| Theam_0389 | pyridoxal phosphate biosynthetic protein PdxJ | *pdxJ* | YP_004151003.1 | 27 | **0.22** |
| Theam_0392 | acyl carrier protein | *acp* | YP_004151006.1 | 9 | **0.10** |
| Theam_0393 | 3-oxoacyl-(acyl-carrier-protein) reductase | *fabG* | YP_004151007.1 | 26 | **0.07** |
| Theam_0397 | hypothetical protein |  | YP_004151011.1 | 24 | **0.12** |
| Theam_0398 | phosphodiesterase, MJ0936 family |  | YP_004151012.1 | 18 | **0.08** |
| Theam_0400 | Conserved TM helix repeat-containing protein |  | YP_004151014.1 | 41 | **0.01** |
| Theam_0404 | HI0933 family protein |  | YP_004151017.1 | 57 | **0.01** |
| Theam_0410 | regulatory protein LuxR | *luxR* | YP_004151023.1 | 22 | **0.01** |
| Theam_0412 | thymidylate kinase | *kthY* | YP_004151025.1 | 24 | **0.05** |
| Theam_0414 | TrkA-C domain protein |  | YP_004151027.1 | 18 | **0.01** |
| Theam_0415 | hypothetical protein |  | YP_004151028.1 | 23 | **0.01** |
| Theam_0417 | PSP1 domain protein |  | YP_004151030.1 | 35 | **0.01** |
| Theam_0418 | hypothetical protein |  | YP_004151031.1 | 52 | **0.19** |
| Theam_0423 | NapA nitrate reductase periplasmic molybdopterin oxidoreductase | *napA* | YP_004151036.1 | 101 | **0.10** |
| Theam_0429 | arginine biosynthesis bifunctional protein ArgJ | *argJ* | YP_004151042.1 | 41 | **0.20** |
| Theam_0430 | tRNA (guanine-N1-)-methyltransferase | *trmD* | YP_004151043.1 | 41 | **0.01** |
| Theam_0431 | cysteine synthase | *cysK* | YP_004151044.1 | 33 | **0.10** |
| Theam_0433 | hypothetical protein |  | YP_004151046.1 | 15 | **0.04** |
| Theam_0436 | tryptophan synthase, alpha subunit | *trpA* | YP_004151049.1 | 29 | **0.20** |
| Theam_0437 | twin-arginine translocation protein, TatA/E family subunit | *tatAE* | YP_004151050.1 | 9 | **0.70** |
| Theam_0439 | transcription termination factor Rho | *rho* | YP_004151052.1 | 48 | **0.04** |
| Theam_0440 | phosphoheptose isomerase | *gmhA* | YP_004151053.1 | 22 | **0.09** |
| Theam_0441 | predicted fructose-bisphosphate aldolase | *fba* | YP_004151054.1 | 28 | **1.71** |
| Theam_0442 | Radical SAM domain protein |  | YP_004151055.1 | 63 | **0.00** |
| Theam_0445 | ABC transporter related for Fe-S assembly | *sufC* | YP_004151058.1 | 27 | **0.14** |
| Theam_0446 | SufBD protein for Fe-S assembly | *sufB* | YP_004151059.1 | 35 | **0.08** |
| Theam_0447 | Putative Superoxide reductase (Desulfoferrodoxin ferrous iron-binding region) | *sor* | YP_004151060.1 | 13 | **0.15** |
| Theam_0449 | Ppx/GppA phosphatase | *gppA* | YP_004151062.1 | 34 | **0.04** |
| Theam_0450 | nucleotide sugar dehydrogenase UDP glucose type | *udg* | YP_004151063.1 | 49 | **0.06** |
| Theam_0452 | 2,5-diamino-6-hydroxy-4-(5-phosphoribosylamino)pyrimidine 1-reductase | *ribD* | YP_004151065.1 | 24 | **0.13** |
| Theam_0456 | regulatory protein ArsR | *arsR* | YP_004151069.1 | 12 | **0.08** |
| Theam_0459 | ketol-acid reductoisomerase | *ilvC* | YP_004151072.1 | 37 | **0.71** |
| Theam_0460 | acetolactate synthase, small subunit | *acolacS* | YP_004151073.1 | 20 | **0.05** |
| Theam_0461 | acetolactate synthase, large subunit, biosynthetic type | *acolacL* | YP_004151074.1 | 64 | **0.18** |
| Theam_0464 | KpsF/GutQ family protein | *kpsF* | YP_004151077.1 | 29 | **0.03** |
| Theam_0467 | [Glutamate--ammonia-ligase] adenylyltransferase | *glnE* | YP_004151080.1 | 102 | **0.05** |
| Theam_0470 | ribosomal protein L34 | *rpmH* | YP_004151083.1 | 7 | **0.03** |
| Theam_0473 | membrane protein insertase, YidC/Oxa1 family | *yidC* | YP_004151086.1 | 55 | **0.01** |
| Theam_0478 | NADH dehydrogenase (quinone) Ni-Fe subunit III | *echE* | YP_004151091.1 | 41 | **0.02** |
| Theam_0480 | NADH ubiquinone oxidoreductase kDa subunit | *echC* | YP_004151093.1 | 20 | **0.02** |
| Theam_0481 | respiratory-chain NADH dehydrogenase subunit 1 | *echB* | YP_004151094.1 | 31 | **0.01** |
| Theam_0483 | cyclic nucleotide-binding |  | YP_004151096.1 | 19 | **0.01** |
| Theam_0484 | nickel-dependent hydrogenase large subunit | *hydA* | YP_004151097.1 | 47 | **0.01** |
| Theam_0485 | NADH ubiquinone oxidoreductase 20 kDa subunit | *fqhD* | YP_004151098.1 | 28 | **0.01** |
| Theam_0487 | cytochrome-c3 hydrogenase alpha chain | *hydB* | YP_004151100.1 | 41 | **0.02** |
| Theam_0488 | pantetheine-phosphate adenylyltransferase | *coaD* | YP_004151101.1 | 19 | **0.04** |
| Theam_0489 | Aldehyde Dehydrogenase | *ald* | YP_004151102.1 | 52 | **0.28** |
| Theam_0490 | metallophosphoesterase |  | YP_004151103.1 | 26 | **0.01** |
| Theam_0491 | tRNA synthetase class II (D K and N) |  | YP_004151104.1 | 35 | **0.03** |
| Theam_0492 | 3-octaprenyl-4-hydroxybenzoate carboxy-lyase | *ubiX* | YP_004151105.1 | 21 | **0.08** |
| Theam_0493 | acetylglutamate kinase | *argB* | YP_004151106.1 | 32 | **0.12** |
| Theam_0494 | cytochrome bd ubiquinol oxidase subunit I | *cydA* | YP_004151107.1 | 52 | **0.01** |
| Theam_0495 | cytochrome d ubiquinol oxidase, subunit II | *cydB* | YP_004151108.1 | 44 | **0.01** |
| Theam_0500 | RNA chaperone Hfq | *hfq* | YP_004151113.1 | 9 | **0.05** |
| Theam_0502 | glutamyl-tRNA synthetase | *gltXb* | YP_004151115.1 | 56 | **0.08** |
| Theam_0505 | Uroporphyrinogen III synthase HEM4 |  | YP_004151118.1 | 27 | **0.01** |
| Theam_0506 | porphobilinogen deaminase | *hemC* | YP_004151119.1 | 35 | **0.14** |
| Theam_0507 | glutamyl-tRNA reductase | *hemA* | YP_004151120.1 | 47 | **0.00** |
| Theam_0509 | 3-dehydroquinate dehydratase, type I | *aroD* | YP_004151122.1 | 28 | **0.14** |
| Theam_0510 | protein of unknown function DUF190 |  | YP_004151123.1 | 13 | **0.02** |
| Theam_0511 | type IV-A pilus assembly ATPase PilB | *pilB* | YP_004151124.1 | 64 | **0.01** |
| Theam_0512 | hypothetical protein |  | YP_004151125.1 | 24 | **0.14** |
| Theam_0514 | 3-phosphoshikimate 1-carboxyvinyltransferase | *aroA* | YP_004151127.1 | 47 | **0.08** |
| Theam_0520 | Porphobilinogen synthase | *hemC* | YP_004151133.1 | 37 | **0.87** |
| Theam_0521 | hypothetical protein |  | YP_004151134.1 | 13 | **0.30** |
| Theam_0522 | beta-lactamase domain-containing protein |  | YP_004151135.1 | 31 | **0.07** |
| Theam_0526 | acetylornithine and succinylornithine aminotransferase | *argD* | YP_004151139.1 | 43 | **0.11** |
| Theam_0534 | dihydroxy-acid dehydratase | *ilvD* | YP_004151147.1 | 59 | **0.17** |
| Theam_0537 | peptidase M22 glycoprotease | *pM22* | YP_004151150.1 | 21 | **0.02** |
| Theam_0542 | hypothetical protein |  | YP_004151155.1 | 6 | **0.05** |
| Theam_0544 | hypothetical protein |  | YP_004151157.1 | 74 | **0.01** |
| Theam_0548 | acetate/CoA ligase - Acetyl-CoA Synthase | *acsA* | YP_004151161.1 | 71 | **0.14** |
| Theam_0549 | excinuclease ABC, B subunit | *uvrb* | YP_004151162.1 | 77 | **0.00** |
| Theam_0551 | Tetratricopeptide TPR_1 repeat-containing protein |  | YP_004151164.1 | 104 | **0.02** |
| Theam_0552 | protein of unknown function DUF507 |  | YP_004151165.1 | 23 | **0.01** |
| Theam_0553 | hypothetical protein |  | YP_004151166.1 | 42 | **0.01** |
| Theam_0566 | Radical SAM domain protein |  | YP_004151179.1 | 25 | **0.02** |
| Theam_0569 | 1-deoxy-D-xylulose 5-phosphate reductoisomerase | *Dxr* | YP_004151182.1 | 41 | **0.04** |
| Theam_0570 | histidyl-tRNA synthetase | *hisS1* | YP_004151183.1 | 47 | **0.04** |
| Theam_0571 | phosphoribosyltransferase |  | YP_004151184.1 | 24 | **0.11** |
| Theam_0574 | hypothetical protein |  | YP_004151187.1 | 32 | **0.04** |
| Theam_0575 | glycosyl transferase family 2 |  | YP_004151188.1 | 111 | **0.01** |
| Theam_0576 | Methyltransferase type 12 |  | YP_004151189.1 | 30 | **0.01** |
| Theam_0577 | glucose-1-phosphate thymidylyltransferase | *rmlA* | YP_004151190.1 | 33 | **0.05** |
| Theam_0578 | dTDP-4-dehydrorhamnose 3,5-epimerase | *rmlC* | YP_004151191.1 | 21 | **0.06** |
| Theam_0579 | dTDP-glucose 4,6-dehydratase | *rmlB* | YP_004151192.1 | 37 | **0.01** |
| Theam_0581 | histone deacetylase superfamily |  | YP_004151194.1 | 33 | **0.03** |
| Theam_0582 | ribonuclease R | *3xrn* | YP_004151195.1 | 82 | **0.07** |
| Theam_0586 | enolase | *eno* | YP_004151199.1 | 47 | **1.09** |
| Theam_0589 | succinyldiaminopimelate transaminase |  | YP_004151202.1 | 44 | **0.02** |
| Theam_0590 | hypothetical protein |  | YP_004151203.1 | 28 | **0.14** |
| Theam_0591 | alkyl hydroperoxide reductase/ Thiol specific antioxidant/ Mal allergen |  | YP_004151204.1 | 18 | **0.04** |
| Theam_0592 | ribosomal protein S20 | *S20* | YP_004151205.1 | 11 | **0.20** |
| Theam_0593 | thiamine-phosphate pyrophosphorylase | *thiE* | YP_004151206.1 | 24 | **0.03** |
| Theam_0599 | glycosyl transferase family 9 |  | YP_004151212.1 | 33 | **0.00** |
| Theam_0601 | nucleotide sugar dehydrogenase |  | YP_004151214.1 | 49 | **0.04** |
| Theam_0602 | malate dehydrogenase, NAD-dependent | *mdh* | YP_004151215.1 | 34 | **0.41** |
| Theam_0603 | Fumarate hydratase, Fe-S type, tartrate/fumarate subfamily, alpha subunit | *fumA* | YP_004151216.1 | 31 | **0.27** |
| Theam_0605 | 1-hydroxy-2-methyl-2-(E)-butenyl 4-diphosphate synthase | *ispG* | YP_004151218.1 | 38 | **0.03** |
| Theam_0607 | diguanylate cyclase | *ggdef* | YP_004151220.1 | 42 | **0.04** |
| Theam_0609 | homocysteine S-methyltransferase | *metH* | YP_004151222.1 | 90 | **0.04** |
| Theam_0610 | adenine phosphoribosyltransferase | *apt* | YP_004151223.1 | 20 | **1.25** |
| Theam_0611 | 3-oxoacyl-[acyl-carrier-protein] synthase 2 |  | YP_004151224.1 | 44 | **0.12** |
| Theam_0612 | ribonuclease III |  | YP_004151225.1 | 27 | **0.02** |
| Theam_0613 | maf protein | *maf* | YP_004151226.1 | 21 | **0.18** |
| Theam_0614 | hypothetical protein |  | YP_004151227.1 | 27 | **0.04** |
| Theam_0616 | 2C-methyl-D-erythritol 2,4-cyclodiphosphate synthase |  | YP_004151229.1 | 18 | **0.01** |
| Theam_0617 | beta-hydroxyacyl-(acyl-carrier-protein) dehydratase FabZ | *fabZ* | YP_004151230.1 | 17 | **0.28** |
| Theam_0618 | acyl-[acyl-carrier-protein]--UDP-N-acetylglucosamine O-acyltransferase | *lpxA* | YP_004151231.1 | 28 | **0.09** |
| Theam_0620 | Nucleoside-triphosphatase |  | YP_004151233.1 | 20 | **0.02** |
| Theam_0621 | Hsp33 protein |  | YP_004151234.1 | 37 | **0.24** |
| Theam_0622 | peptidase zinc-dependent |  | YP_004151235.1 | 19 | **0.06** |
| Theam_0624 | Purine-nucleoside phosphorylase |  | YP_004151237.1 | 29 | **0.03** |
| Theam_0625 | hypothetical protein |  | YP_004151238.1 | 63 | **0.00** |
| Theam_0626 | ammonium transporter |  | YP_004151239.1 | 12 | **0.10** |
| Theam_0629 | glutamine synthetase, type I | *glnA* | YP_004151242.1 | 53 | **0.95** |
| Theam_0631 | diaminopimelate decarboxylase | *lysA* | YP_004151243.1 | 46 | **0.05** |
| Theam_0633 | Prephenate dehydrogenase |  | YP_004151245.1 | 32 | **0.10** |
| Theam_0634 | class II aldolase/adducin family protein |  | YP_004151246.1 | 21 | **0.04** |
| Theam_0635 | histidine kinase HAMP region domain protein |  | YP_004151247.1 | 37 | **0.02** |
| Theam_0636 | hypothetical protein |  | YP_004151248.1 | 33 | **0.02** |
| Theam_0638 | protein of unknown function DUF178 |  | YP_004151249.1 | 32 | **0.01** |
| Theam_0640 | rfaE bifunctional protein | *rfaE* | YP_004151251.1 | 36 | **0.04** |
| Theam_0642 | isoleucyl-tRNA synthetase | *ileS* | YP_004151253.1 | 108 | **0.08** |
| Theam_0644 | Rhodanese domain protein |  | YP_004151255.1 | 15 | **0.02** |
| Theam_0645 | phosphoglycerate mutase, 2,3-bisphosphoglycerate-independent | *pgm* | YP_004151256.1 | 58 | **0.03** |
| Theam_0646 | tRNA pseudouridine synthase A | *hisT_truA* | YP_004151257.1 | 30 | **0.01** |
| Theam_0648 | glutamate--cysteine ligase GCS2 |  | YP_004151259.1 | 40 | **0.02** |
| Theam_0649 | gamma-glutamyltransferase |  | YP_004151260.1 | 54 | **0.04** |
| Theam_0651 | protein-P-II uridylyltransferase | *glnD* | YP_004151262.1 | 102 | **0.01** |
| Theam_0654 | UDP-glucose 4-epimerase | *galE* | YP_004151265.1 | 36 | **0.02** |
| Theam_0655 | triosephosphate isomerase | *tim* | YP_004151266.1 | 27 | **0.10** |
| Theam_0656 | ATP-dependent Clp protease, ATP-binding subunit ClpX | *clpX* | YP_004151267.1 | 46 | **0.07** |
| Theam_0657 | N-(5'phosphoribosyl)anthranilate isomerase (PRAI) |  | YP_004151268.1 | 23 | **0.10** |
| Theam_0658 | Ferritin Dps family protein |  | YP_004151269.1 | 19 | **0.09** |
| Theam_0661 | aspartyl-tRNA synthetase | *aspSb* | YP_004151271.1 | 68 | **0.11** |
| Theam_0663 | 3'(2'),5'-bisphosphate nucleotidase | *cysQ* | YP_004151273.1 | 29 | **0.07** |
| Theam_0666 | sulfate adenylyltransferase | *aps* | YP_004151276.1 | 44 | **0.03** |
| Theam_0671 | glycosyl transferase group 1 |  | YP_004151281.1 | 40 | **0.00** |
| Theam_0677 | RNA polymerase-binding protein DksA |  | YP_004151286.1 | 15 | **0.02** |
| Theam_0678 | aminotransferase class I and II |  | YP_004151287.1 | 44 | **0.17** |
| Theam_0683 | EAL domain protein |  | YP_004151292.1 | 75 | **0.03** |
| Theam_0686 | NAD-dependent glycerol-3-phosphate dehydrogenase domain protein |  | YP_004151295.1 | 35 | **0.03** |
| Theam_0688 | phosphate transport system regulatory protein PhoU | *phoU* | YP_004151297.1 | 27 | **0.21** |
| Theam_0691 | NADPH-dependent FMN reductase | *fmnr* | YP_004151300.1 | 21 | **0.03** |
| Theam_0692 | ribosome-associated GTPase EngA | *engA* | YP_004151301.1 | 55 | **0.03** |
| Theam_0693 | hypothetical protein |  | YP_004151302.1 | 24 | **0.04** |
| Theam_0694 | hypothetical protein |  | YP_004151303.1 | 21 | **0.04** |
| Theam_0701 | lysine 2,3-aminomutase YodO family protein | *yodO* | YP_004151310.1 | 43 | **0.04** |
| Theam_0703 | pseudogene |  |  | 18 | **0.05** |
| Theam_0704 | histidinol-phosphate aminotransferase | *hisC* | YP_004151312.1 | 42 | **0.09** |
| Theam_0709 | 3-methyl-2-oxobutanoate hydroxymethyltransferase | *panB* | YP_004151317.1 | 29 | **0.07** |
| Theam_0710 | ribosome small subunit-dependent GTPase A | *rsgA* | YP_004151318.1 | 34 | **0.03** |
| Theam_0712 | ketose-bisphosphate aldolase class-II |  | YP_004151320.1 | 53 | **0.22** |
| Theam_0714 | L-aspartate oxidase | *nadB* | YP_004151322.1 | 57 | **0.01** |
| Theam_0718 | CTP synthase | *PyrG* | YP_004151326.1 | 60 | **0.04** |
| Theam_0719 | 3-deoxy-D-manno-octulosonate cytidylyltransferase | *kdsB* | YP_004151327.1 | 27 | **0.02** |
| Theam_0720 | phosphoribosylformylglycinamidine synthase I | *fgams* | YP_004151328.1 | 25 | **0.14** |
| Theam_0721 | phosphoribosylformylglycinamidine synthase, purS | *purS* | YP_004151329.1 | 10 | **0.16** |
| Theam_0725 | RNA polymerase sigma factor RpoD | *rpoD* | YP_004151333.1 | 66 | **0.01** |
| Theam_0727 | Peroxiredoxin |  | YP_004151335.1 | 22 | **0.76** |
| Theam_0730 | Fumarate hydratase, Fe-S type, tartrate/fumarate subfamily, beta subunit | *fumB* | YP_004151338.1 | 21 | **0.25** |
| Theam_0734 | NADH dehydrogenase I, D subunit | *nuoD* | YP_004151342.1 | 45 | **0.02** |
| Theam_0736 | NADH-quinone oxidoreductase, F subunit | *nuoF* | YP_004151344.1 | 46 | **0.00** |
| Theam_0737 | NADH:ubiquinone oxidoreductase, subunit G, iron-sulphur binding | *nuoG* | YP_004151345.1 | 80 | **0.06** |
| Theam_0738 | putative transcriptional regulator, Crp/Fnr family |  | YP_004151346.1 | 18 | **0.04** |
| Theam_0746 | L-seryl-tRNA selenium transferase | *selA* | YP_004151354.1 | 52 | **0.01** |
| Theam_0747 | TonB-dependent receptor |  | YP_004151355.1 | 76 | **0.00** |
| Theam_0751 | Peptidoglycan-binding lysin domain |  | YP_004151359.1 | 27 | **0.14** |
| Theam_0752 | hypothetical protein |  | YP_004151360.1 | 13 | **0.10** |
| Theam_0753 | acriflavin resistance protein |  | YP_004151361.1 | 121 | **0.03** |
| Theam_0754 | efflux transporter, RND family, MFP subunit | *mfp* | YP_004151362.1 | 42 | **0.05** |
| Theam_0755 | glutamine amidotransferase class-I | *gmps* | YP_004151363.1 | 26 | **0.07** |
| Theam_0756 | peptidase U62 modulator of DNA gyrase | *tldD* | YP_004151364.1 | 50 | **0.09** |
| Theam_0757 | NAD+ synthetase | *nadE* | YP_004151365.1 | 31 | **0.11** |
| Theam_0758 | Nitrilase/cyanide hydratase and apolipoprotein N-acyltransferase |  | YP_004151366.1 | 32 | **0.09** |
| Theam_0763 | PhoH family protein |  | YP_004151371.1 | 36 | **0.08** |
| Theam_0765 | SMC domain protein |  | YP_004151373.1 | 105 | **0.00** |
| Theam_0768 | Radical SAM domain protein |  | YP_004151376.1 | 35 | **0.02** |
| Theam_0770 | phosphoesterase RecJ domain protein |  | YP_004151378.1 | 37 | **0.10** |
| Theam_0772 | peptidase U62 modulator of DNA gyrase | *tldD* | YP_004151380.1 | 48 | **0.11** |
| Theam_0773 | thymidylate synthase, flavin-dependent |  | YP_004151381.1 | 23 | **0.04** |
| Theam_0778 | NHL repeat containing protein |  | YP_004151386.1 | 29 | **0.04** |
| Theam_0779 | hypothetical protein |  | YP_004151387.1 | 27 | **0.01** |
| Theam_0784 | hypothetical protein |  | YP_004151392.1 | 50 | **0.00** |
| Theam_0785 | molybdenum cofactor synthesis domain protein |  | YP_004151393.1 | 43 | **0.01** |
| Theam_0786 | molybdopterin binding domain |  | YP_004151394.1 | 38 | **0.02** |
| Theam_0790 | mannose-1-phosphate guanylyltransferase/mannose-6-phosphate isomerase |  | YP_004151398.1 | 53 | **0.03** |
| Theam_0797 | nucleotide sugar dehydrogenase |  | YP_004151405.1 | 50 | **0.03** |
| Theam_0800 | DNA polymerase I | *dna2* | YP_004151408.1 | 94 | **0.01** |
| Theam_0804 | putative lipoprotein |  | YP_004151412.1 | 16 | **0.03** |
| Theam_0812 | DNA helicase |  | YP_004151420.1 | 83 | **0.02** |
| Theam_0814 | hypothetical protein |  | YP_004151422.1 | 31 | **0.01** |
| Theam_0815 | Nucleoside-diphosphate kinase |  | YP_004151423.1 | 16 | **0.20** |
| Theam_0820 | protein-export membrane protein SecD | *secD* | YP_004151428.1 | 61 | **0.00** |
| Theam_0824 | UDP-N-acetylglucosamine 1-carboxyvinyltransferase | *murA* | YP_004151432.1 | 47 | **0.00** |
| Theam_0825 | ATP phosphoribosyltransferase | *hisG* | YP_004151433.1 | 24 | **0.06** |
| Theam_0826 | formyltetrahydrofolate deformylase | *PurU* | YP_004151434.1 | 33 | **0.04** |
| Theam_0827 | glucosamine/fructose-6-phosphate aminotransferase, isomerizing | *glmS* | YP_004151435.1 | 68 | **0.05** |
| Theam_0828 | tRNA modification GTPase TrmE |  | YP_004151436.1 | 52 | **0.04** |
| Theam_0830 | protein of unknown function DUF583 |  | YP_004151438.1 | 13 | **0.20** |
| Theam_0836 | HAD-superfamily hydrolase, subfamily IIA |  | YP_004151444.1 | 29 | **0.12** |
| Theam_0838 | GTP-binding protein YchF | *ychF* | YP_004151446.1 | 41 | **0.02** |
| Theam_0843 | response regulator receiver | *cheY* | YP_004151451.1 | 15 | **0.08** |
| Theam_0844 | response regulator receiver | *cheV* | YP_004151452.1 | 36 | **0.03** |
| Theam_0845 | chemotaxis sensory transducer | *mcp* | YP_004151453.1 | 72 | **0.09** |
| Theam_0846 | CheW domain protein | *cheW* | YP_004151454.1 | 19 | **0.02** |
| Theam_0847 | ATP-binding region ATPase domain protein - histidine kinase | *cheA* | YP_004151455.1 | 74 | **0.03** |
| Theam_0848 | putative myosin-2 heavy chain, non muscle | *cheZ* | YP_004151456.1 | 20 | **0.07** |
| Theam_0849 | cell division protein FtsZ | *ftsZ* | YP_004151457.1 | 39 | **0.04** |
| Theam_0852 | D-alanine/D-alanine ligase |  | YP_004151460.1 | 32 | **0.01** |
| Theam_0854 | UDP-N-acetylmuramyl-tripeptide synthetase | *murE* | YP_004151462.1 | 53 | **0.00** |
| Theam_0855 | hypothetical protein |  | YP_004151463.1 | 11 | **0.04** |
| Theam_0856 | hypothetical protein |  | YP_004151464.1 | 10 | **0.02** |
| Theam_0862 | outer membrane efflux protein |  | YP_004151470.1 | 48 | **0.01** |
| Theam_0873 | DNA ligase, NAD-dependent | *dnlj* | YP_004151481.1 | 82 | **0.05** |
| Theam_0876 | molybdenum cofactor biosynthesis protein C | *moa* | YP_004151484.1 | 33 | **0.36** |
| Theam_0877 | hypothetical protein |  | YP_004151485.1 | 21 | **0.02** |
| Theam_0878 | molybdopterin biosynthesis MoaE protein | *moaE* | YP_004151486.1 | 13 | **0.01** |
| Theam_0884 | signal recognition particle-docking protein FtsY | *ftsY* | YP_004151492.1 | 54 | **0.02** |
| Theam_0885 | anthranilate phosphoribosyltransferase | *trpD* | YP_004151493.1 | 38 | **0.04** |
| Theam_0887 | metalloendopeptidase, glycoprotease family | *gcp* | YP_004151495.1 | 35 | **0.03** |
| Theam_0889 | transglutaminase domain-containing protein |  | YP_004151497.1 | 72 | **0.00** |
| Theam_0890 | phosphoglucosamine mutase | *glmM* | YP_004151498.1 | 49 | **0.07** |
| Theam_0893 | dihydropteroate synthase | *dhps* | YP_004151501.1 | 44 | **0.01** |
| Theam_0894 | Polynucleotide adenylyltransferase region |  | YP_004151502.1 | 98 | **0.01** |
| Theam_0896 | general secretion pathway protein D | *gspD* | YP_004151504.1 | 71 | **0.04** |
| Theam_0903 | type II and III secretion system protein | *gspC* | YP_004151511.1 | 72 | **0.02** |
| Theam_0906 | reverse gyrase | *rgy* | YP_004151514.1 | 137 | **0.04** |
| Theam_0907 | iron-containing alcohol dehydrogenase |  | YP_004151515.1 | 41 | **0.09** |
| Theam_0909 | transcription antitermination factor NusB | *nusB* | YP_004151517.1 | 16 | **0.03** |
| Theam_0910 | 6,7-dimethyl-8-ribityllumazine synthase | *ribH* | YP_004151518.1 | 17 | **0.27** |
| Theam_0911 | enoyl-(acyl-carrier-protein) reductase II | *fabK* | YP_004151519.1 | 34 | **0.07** |
| Theam_0912 | 3-oxoacyl-(acyl-carrier-protein) synthase III | *fabH* | YP_004151520.1 | 34 | **0.05** |
| Theam_0913 | fatty acid/phospholipid synthesis protein PlsX | *plsX* | YP_004151521.1 | 36 | **0.00** |
| Theam_0919 | methylenetetrahydrofolate reductase F | *metF* | YP_004151527.1 | 32 | **0.04** |
| Theam_0920 | undecaprenyl diphosphate synthase | *uppS* | YP_004151528.1 | 27 | **0.05** |
| Theam_0922 | hydrogenase expression/formation protein HypE | *hypE* | YP_004151530.1 | 35 | **0.11** |
| Theam_0924 | hydrogenase expression/formation protein HypD | *hypD* | YP_004151532.1 | 39 | **0.06** |
| Theam_0936 | hypothetical protein |  | YP_004151544.1 | 38 | **0.05** |
| Theam_0938 | Orn/DAP/Arg decarboxylase 2 |  | YP_004151546.1 | 51 | **0.02** |
| Theam_0940 | hypothetical protein |  | YP_004151548.1 | 34 | **0.10** |
| Theam_0942 | phosphoribosylamine/glycine ligase | *purD* | YP_004151550.1 | 46 | **0.08** |
| Theam_0948 | histidine triad (HIT) protein |  | YP_004151556.1 | 19 | **0.18** |
| Theam_0953 | Domain of unkown function DUF1786 putative pyruvate format-lyase activating enzyme |  | YP_004151561.1 | 37 | **0.01** |
| Theam_0954 | cell division protein FtsZ | *ftsZ* | YP_004151562.1 | 39 | **0.03** |
| Theam_0955 | Nicotinamidase | *pncA* | YP_004151563.1 | 21 | **0.10** |
| Theam_0956 | hypothetical protein |  | YP_004151564.1 | 33 | **0.22** |
| Theam_0962 | peptidase M16 domain protein |  | YP_004151570.1 | 46 | **0.02** |
| Theam_0963 | hypothetical protein |  | YP_004151571.1 | 48 | **0.00** |
| Theam_0964 | ADP-ribosylation/Crystallin J1 |  | YP_004151572.1 | 35 | **0.03** |
| Theam_0965 | hypothetical protein |  | YP_004151573.1 | 126 | **0.01** |
| Theam_0969 | fumarate |  | YP_004151577.1 | 42 | **0.01** |
| Theam_0970 | Radical SAM domain protein |  | YP_004151578.1 | 42 | **0.01** |
| Theam_0972 | phosphoenolpyruvate synthase water dikinase | *ppsA* | YP_004151580.1 | 91 | **0.08** |
| Theam_0973 | D-isomer specific 2-hydroxyacid dehydrogenase NAD-binding |  | YP_004151581.1 | 38 | **0.04** |
| Theam_0974 | phosphoglucose isomerase | *pgi* | YP_004151582.1 | 45 | **0.03** |
| Theam_0984 | malonyl CoA-acyl carrier protein transacylase | *fabD* | YP_004151592.1 | 34 | **0.21** |
| Theam_0985 | ribonucleoside-diphosphate reductase, adenosylcobalamin-dependent |  | YP_004151593.1 | 63 | **0.01** |
| Theam_0990 | twitching motility protein | *pilT* | YP_004151598.1 | 40 | **0.03** |
| Theam_0998 | Glutamate synthase (NADPH) | *gltA* | YP_004151606.1 | 55 | **0.17** |
| Theam_1000 | NAD – Nitrite reductase (FAD-dependent pyridine nucleotide-disulphide oxidoreductase) | *nirA* | YP_004151608.1 | 47 | **0.20** |
| Theam_1001 | glutamine amidotransferase class-II |  | YP_004151609.1 | 42 | **0.12** |
| Theam_1002 | glutamate synthase alpha subunit domain protein | *glt* | YP_004151610.1 | 29 | **0.24** |
| Theam_1003 | tryptophan synthase, beta subunit | *trpB* | YP_004151611.1 | 44 | **0.10** |
| Theam_1005 | tyrosyl-tRNA synthetase | *tyrS* | YP_004151613.1 | 47 | **0.07** |
| Theam_1008 | lysyl-tRNA synthetase | *lysSb* | YP_004151616.1 | 61 | **0.11** |
| Theam_1011 | Mammalian cell entry related domain protein |  | YP_004151619.1 | 58 | **0.00** |
| Theam_1013 | ??fumarate reductase/succinate dehydrogenase flavoprotein domain protein | *fum?* | YP_004151621.1 | 20 | **0.26** |
| Theam_1017 | adenylosuccinate lyase | *purB* | YP_004151625.1 | 51 | **0.25** |
| Theam_1020 | molybdopterin oxidoreductase fdhA hydrogenase family | *fdhA* | YP_004151628.1 | 76 | **0.04** |
| Theam_1021 | ATP-citrate (pro-S-)-lyase subunit alpha | *aclA* | YP_004151629.1 | 68 | **2.03** |
| Theam_1022 | ATP-citrate (pro-S-)-lyase subunit beta | *aclB* | YP_004151630.1 | 49 | **0.64** |
| Theam_1023 | isocitrate dehydrogenase/2-oxoglutarate carboxylase , NADP-dependent | *idh2* | YP_004151631.1 | 83 | **1.24** |
| Theam_1024 | aconitate hydratase | *acnB* | YP_004151632.1 | 71 | **0.59** |
| Theam_1027 | methyl-accepting chemotaxis protein |  | YP_004151635.1 | 38 | **0.04** |
| Theam_1028 | hypothetical protein |  | YP_004151636.1 | 42 | **0.12** |
| Theam_1029 | hypothetical protein |  | YP_004151637.1 | 75 | **0.63** |
| Theam_1035 | phosphopantothenoylcysteine decarboxylase/phosphopantothenate/cysteine ligase | *coaBC_dfp* | YP_004151643.1 | 42 | **0.01** |
| Theam_1036 | RNA methyltransferase, TrmH family, group 3 |  | YP_004151644.1 | 26 | **0.02** |
| Theam_1041 | carbohydrate kinase, YjeF related protein |  | YP_004151649.1 | 57 | **0.10** |
| Theam_1042 | peptide deformylase |  | YP_004151650.1 | 20 | **0.07** |
| Theam_1043 | Xylose isomerase domain-containing protein TIM barrel |  | YP_004151651.1 | 28 | **0.02** |
| Theam_1045 | Phosphoribosyl-AMP cyclohydrolase |  | YP_004151653.1 | 13 | **0.11** |
| Theam_1053 | tRNA 2-selenouridine synthase |  | YP_004151661.1 | 40 | **0.01** |
| Theam_1055 | pyruvate fromate-lyase activating enzyme |  | YP_004151663.1 | 38 | **0.03** |
| Theam_1056 | PEBP family protein |  | YP_004151664.1 | 17 | **0.01** |
| Theam_1062 | heat shock protein Hsp20 |  | YP_004151670.1 | 20 | **0.04** |
| Theam_1063 | PHP domain protein |  | YP_004151671.1 | 65 | **0.02** |
| Theam_1065 | hypothetical protein |  | YP_004151673.1 | 41 | **0.02** |
| Theam_1067 | 3-dehydroquinate synthase | *aroB* | YP_004151675.1 | 37 | **0.32** |
| Theam_1069 | ADP-L-glycero-D-manno-heptose-6-epimerase |  | YP_004151677.1 | 36 | **0.14** |
| Theam_1070 | regulatory protein MerR | *merR* | YP_004151678.1 | 14 | **0.01** |
| Theam_1071 | ATPase AAA-2 domain protein | *clp?* | YP_004151679.1 | 95 | **0.12** |
| Theam_1075 | Methionine adenosyltransferase | *samS* | YP_004151683.1 | 44 | **0.29** |
| Theam_1076 | hypothetical protein |  | YP_004151684.1 | 71 | **0.01** |
| Theam_1078 | hypothetical protein |  | YP_004151686.1 | 30 | **0.02** |
| Theam_1081 | preprotein translocase, SecA subunit | *secA* | YP_004151689.1 | 100 | **0.06** |
| Theam_1083 | protease Do |  | YP_004151691.1 | 52 | **0.05** |
| Theam_1087 | MotA/TolQ/ExbB proton channel | *motA* | YP_004151695.1 | 27 | **0.01** |
| Theam_1088 | D-lactate dehydrogenase (cytochrome) |  | YP_004151696.1 | 49 | **0.03** |
| Theam_1090 | polysaccharide export protein |  | YP_004151698.1 | 107 | **0.01** |
| Theam_1095 | branched-chain amino acid aminotransferase | *ilvE_I* | YP_004151703.1 | 34 | **0.32** |
| Theam_1098 | general secretion pathway protein G | *gspG* | YP_004151706.1 | 16 | **0.02** |
| Theam_1100 | hypothetical protein |  | YP_004151708.1 | 14 | **0.27** |
| Theam_1106 | uroporphyrinogen decarboxylase | *hemE* | YP_004151713.1 | 39 | **0.14** |
| Theam_1107 | exodeoxyribonuclease III Xth | *xth* | YP_004151714.1 | 30 | **0.01** |
| Theam_1109 | protein-L-isoaspartate(D-aspartate) O-methyltransferase |  | YP_004151716.1 | 30 | **0.03** |
| Theam_1111 | protein of unknown function DUF28 |  | YP_004151718.1 | 27 | **0.01** |
| Theam_1113 | hydrolase, TatD family |  | YP_004151720.1 | 53 | **0.02** |
| Theam_1115 | arginyl-tRNA synthetase | *argS* | YP_004151722.1 | 62 | **0.09** |
| Theam_1116 | [NiFe] hydrogenase maturation protein HypF | *hypF* | YP_004151723.1 | 83 | **0.01** |
| Theam_1121 | hydrogenase (NiFe) small subunit HydA | *hynA* | YP_004151728.1 | 39 | **0.03** |
| Theam_1122 | nickel-dependent hydrogenase large subunit | *hynB* | YP_004151729.1 | 65 | **0.27** |
| Theam_1123 | Ni/Fe-hydrogenase, b-type cytochrome subunit | *hynC* | YP_004151730.1 | 26 | **0.01** |
| Theam_1124 | hydrogenase 2 maturation protease | *hupD* | YP_004151731.1 | 20 | **0.02** |
| Theam_1128 | hydrogenase accessory protein HypB | *hypB* | YP_004151735.1 | 28 | **0.24** |
| Theam_1130 | outer membrane efflux protein |  | YP_004151737.1 | 51 | **0.00** |
| Theam_1133 | flavodoxin/nitric oxide synthase not related to nitrogen metabolism |  | YP_004151740.1 | 45 | **0.09** |
| Theam_1138 | glutamyl-tRNA(Gln) amidotransferase, B subunit | *gatB* | YP_004151743.1 | 55 | **0.20** |
| Theam_1141 | DNA gyrase, A subunit | *gyrA* | YP_004151746.1 | 91 | **0.08** |
| Theam_1142 | seryl-tRNA synthetase | *serS* | YP_004151747.1 | 49 | **0.09** |
| Theam_1150 | transcriptional regulator domain-containing protein |  | YP_004151755.1 | 25 | **0.01** |
| Theam_1154 | 3-deoxy-D-manno-octulosonate 8-phosphate phosphatase, YrbI family |  | YP_004151759.1 | 17 | **0.02** |
| Theam_1156 | Cytochrome-c peroxidase | *ccpA* | YP_004151761.1 | 45 | **0.02** |
| Theam_1157 | dethiobiotin synthase | *bioD* | YP_004151762.1 | 22 | **0.07** |
| Theam_1162 | flagellin domain protein - flagellin structural protein | *FliC* | YP_004151767.1 | 32 | **1.66** |
| Theam_1165 | glycosyl transferase family 2 |  | YP_004151770.1 | 47 | **0.06** |
| Theam_1166 | riboflavin biosynthesis protein RibD | *ribD* | YP_004151771.1 | 40 | **0.00** |
| Theam_1168 | tryptophanyl-tRNA synthetase | *trpS* | YP_004151773.1 | 41 | **0.10** |
| Theam_1173 | phosphoribosylaminoimidazole-succinocarboxamide synthase | *purC* | YP_004151778.1 | 28 | **0.34** |
| Theam_1174 | amidophosphoribosyltransferase | *purF* | YP_004151779.1 | 51 | **0.04** |
| Theam_1177 | threonyl-tRNA synthetase | *thrS* | YP_004151782.1 | 76 | **0.05** |
| Theam_1178 | translation initiation factor IF-3 | *infC* | YP_004151783.1 | 17 | **0.03** |
| Theam_1180 | ribosomal protein L20 | *rplT* | YP_004151785.1 | 14 | **0.13** |
| Theam_1181 | hypothetical protein |  | YP_004151786.1 | 41 | **0.13** |
| Theam_1183 | 6-pyruvoyl tetrahydropterin synthase and hypothetical protein |  | YP_004151788.1 | 24 | **0.18** |
| Theam_1184 | methyltransferase | *metR* | YP_004151789.1 | 22 | **0.08** |
| Theam_1188 | DNA gyrase, B subunit | *gyrB* | YP_004151793.1 | 91 | **0.03** |
| Theam_1189 | cytidylate kinase | *cmk* | YP_004151794.1 | 24 | **0.06** |
| Theam_1190 | nicotinate phosphoribosyltransferase |  | YP_004151795.1 | 49 | **0.04** |
| Theam_1191 | DNA-(apurinic or apyrimidinic site) lyase |  | YP_004151796.1 | 25 | **0.01** |
| Theam_1192 | 2-dehydro-3-deoxyphosphooctonate aldolase | *kdo* | YP_004151797.1 | 30 | **0.07** |
| Theam_1195 | deoxyribose-phosphate aldolase | *deoC* | YP_004151800.1 | 24 | **0.03** |
| Theam_1197 | GTP-binding protein Era | *era* | YP_004151802.1 | 35 | **0.03** |
| Theam_1201 | hypothetical protein |  | YP_004151806.1 | 9 | **0.15** |
| Theam_1203 | phenylalanyl-tRNA synthetase, alpha subunit | *pheS* | YP_004151808.1 | 39 | **0.10** |
| Theam_1204 | phenylalanyl-tRNA synthetase, beta subunit | *pheTb* | YP_004151809.1 | 89 | **0.18** |
| Theam_1206 | 5-formyltetrahydrofolate cyclo-ligase | *folA* | YP_004151811.1 | 21 | **0.02** |
| Theam_1207 | YmdA/YtgF protein | *hdig* | YP_004151812.1 | 63 | **0.02** |
| Theam_1208 | selenocysteine-specific translation elongation factor | *selB* | YP_004151813.1 | 71 | **0.00** |
| Theam_1211 | phosphoribosylglycinamide formyltransferase 2 | *purT* | YP_004151816.1 | 44 | **0.12** |
| Theam_1213 | Nitrilase/cyanide hydratase and apolipoprotein N-acyltransferase |  | YP_004151818.1 | 28 | **0.03** |
| Theam_1215 | pyrroline-5-carboxylate reductase | *proC* | YP_004151820.1 | 29 | **0.10** |
| Theam_1217 | Phosphomethylpyrimidine kinase |  | YP_004151822.1 | 26 | **0.01** |
| Theam_1218 | sugar-phosphate isomerase, RpiB/LacA/LacB family | *rpiB* | YP_004151823.1 | 17 | **0.07** |
| Theam_1219 | Glycine hydroxymethyltransferase |  | YP_004151824.1 | 46 | **0.20** |
| Theam_1221 | chaperonin GroEL |  | YP_004151826.1 | 59 | **1.37** |
| Theam_1222 | Chaperonin Cpn10 |  | YP_004151827.1 | 11 | **1.03** |
| Theam_1223 | transglutaminase domain-containing protein |  | YP_004151828.1 | 35 | **0.03** |
| Theam_1225 | phosphate-selective porin O and P |  | YP_004151830.1 | 44 | **0.13** |
| Theam_1226 | Hydrogenase Mo catalitic subunit - molydopterin dinucleotide-binding region | *hycB2* | YP_004151831.1 | 131 | **0.12** |
| Theam_1227 | hypothetical protein |  | YP_004151832.1 | 27 | **0.01** |
| Theam_1228 | CBS domain containing protein |  | YP_004151833.1 | 15 | **0.10** |
| Theam_1234 | ATPase-like, ParA/MinD |  | YP_004151839.1 | 32 | **0.18** |
| Theam_1235 | inositol monophosphatase |  | YP_004151840.1 | 28 | **0.13** |
| Theam_1238 | histidine kinase |  | YP_004151843.1 | 34 | **0.01** |
| Theam_1240 | thioesterase superfamily protein |  | YP_004151845.1 | 14 | **0.05** |
| Theam_1241 | hypothetical protein |  | YP_004151846.1 | 23 | **0.01** |
| Theam_1242 | peptide chain release factor 1 | *prfA* | YP_004151847.1 | 41 | **0.04** |
| Theam_1244 | Radical SAM domain protein |  | YP_004151849.1 | 43 | **0.03** |
| Theam_1246 | 2,3,4,5-tetrahydropyridine-2,6-carboxylate N-succinyltransferase |  | YP_004151851.1 | 30 | **0.15** |
| Theam_1247 | methionyl-tRNA synthetase | *metG* | YP_004151852.1 | 61 | **0.09** |
| Theam_1249 | S-Adenosyl homocisteine hydrolase (adenosylhomocysteinase) | *sahH* | YP_004151854.1 | 47 | **0.16** |
| Theam_1250 | UDP-3-O-[3-hydroxymyristoyl] glucosamine N-acyltransferase | *lpxD* | YP_004151855.1 | 36 | **0.03** |
| Theam_1251 | outer membrane chaperone Skp (OmpH) |  | YP_004151856.1 | 20 | **0.01** |
| Theam_1252 | outer membrane protein assembly complex, YaeT protein |  | YP_004151857.1 | 87 | **0.02** |
| Theam_1254 | glyceraldehyde 3-phosphate dehydrogenase, type I | *gapdh* | YP_004151859.1 | 36 | **0.68** |
| Theam_1256 | nicotinate-nucleotide pyrophosphorylase | *nadC* | YP_004151861.1 | 32 | **0.04** |
| Theam_1259 | dephospho-CoA kinase |  | YP_004151864.1 | 21 | **0.02** |
| Theam_1260 | metallophosphoesterase |  | YP_004151865.1 | 30 | **0.04** |
| Theam_1261 | Methylenetetrahydrofolate dehydrogenase (NADP(+)) | *folD* | YP_004151866.1 | 31 | **0.22** |
| Theam_1265 | AMP-dependent synthetase and ligase |  | YP_004151869.1 | 61 | **0.01** |
| Theam_1266 | diaminopimelate epimerase | *DapF* | YP_004151870.1 | 30 | **0.01** |
| Theam_1267 | phage SPO1 DNA polymerase-related protein | *spo1* | YP_004151871.1 | 27 | **0.07** |
| Theam_1270 | CoB--CoM heterodisulfide reductase / Fumarate reductase ascciated protein A? | *hdrA* | YP_004151874.1 | 32 | **0.09** |
| Theam_1271 | fumarate reductase iron-sulfur protein | *frdB1* | YP_004151875.1 | 43 | **0.11** |
| Theam_1272 | fumarate reductase, flavoprotein subunit | *frdB* | YP_004151876.1 | 62 | **0.18** |
| Theam_1273 | fumarate reductase, flavoprotein subunit | *frdA1* | YP_004151877.1 | 63 | **0.07** |
| Theam_1274 | fumarate reductase iron-sulfur protein | *frdA* | YP_004151878.1 | 36 | **0.03** |
| Theam_1275 | CoB--CoM heterodisulfide reductase / Fumarate reductase ascciated protein B? | *hdrB* | YP_004151879.1 | 32 | **0.05** |
| Theam_1276 | outer membrane efflux protein |  | YP_004151880.1 | 50 | **0.02** |
| Theam_1279 | pyruvate kinase | *pvtK* | YP_004151883.1 | 51 | **0.01** |
| Theam_1282 | phosphoglucomutase/phosphomannomutase alpha/beta/alpha domain I |  | YP_004151886.1 | 52 | **0.20** |
| Theam_1283 | Circadian clock protein KaiC central region |  | YP_004151887.1 | 33 | **0.11** |
| Theam_1284 | P450 cytochrome, putative |  | YP_004151888.1 | 16 | **0.07** |
| Theam_1293 | Silent information regulator protein Sir2 |  | YP_004151897.1 | 30 | **0.02** |
| Theam_1296 | putative metal-dependent hydrolase |  | YP_004151900.1 | 26 | **0.06** |
| Theam_1297 | signal recognition particle protein | *ffh* | YP_004151901.1 | 50 | **0.01** |
| Theam_1298 | ribosomal protein S16 | *S16* | YP_004151902.1 | 9 | **0.10** |
| Theam_1299 | hypothetical protein |  | YP_004151903.1 | 9 | **0.20** |
| Theam_1300 | hypothetical protein |  | YP_004151904.1 | 22 | **0.11** |
| Theam_1301 | transaldolase | *talC* | YP_004151905.1 | 24 | **0.50** |
| Theam_1303 | hypothetical protein |  | YP_004151907.1 | 24 | **0.06** |
| Theam_1307 | protein of unknown function DUF77 |  | YP_004151911.1 | 12 | **0.02** |
| Theam_1309 | pantoate/beta-alanine ligase |  | YP_004151913.1 | 32 | **0.02** |
| Theam_1311 | hypothetical protein |  | YP_004151915.1 | 13 | **0.05** |
| Theam_1312 | glutamate 5-kinase | *proB* | YP_004151916.1 | 38 | **0.01** |
| Theam_1313 | GTP-binding protein Obg/CgtA |  | YP_004151917.1 | 38 | **0.03** |
| Theam_1317 | hypothetical protein |  | YP_004151921.1 | 13 | **0.03** |
| Theam_1320 | Ppx/GppA phosphatase |  | YP_004151924.1 | 33 | **0.02** |
| Theam_1321 | Pyrococcus NSR homolog / FAD-dependent pyridine nucleotide-disulphide oxidoreductase CoB-CoM related | *nsr* | YP_004151925.1 | 47 | **0.11** |
| Theam_1323 | fructose-1,6-bisphosphatase I | *fbp* | YP_004151927.1 | 34 | **0.20** |
| Theam_1324 | radical SAM enzyme, Cfr family |  | YP_004151928.1 | 39 | **0.02** |
| Theam_1327 | histidinol dehydrogenase | *hisD* | YP_004151931.1 | 49 | **0.07** |
| Theam_1328 | aspartate carbamoyltransferase | *atc* | YP_004151932.1 | 34 | **0.18** |
| Theam_1330 | dihydroorotase, multifunctional complex type | *pyrC* | YP_004151934.1 | 46 | **0.05** |
| Theam_1331 | periplasmic serine protease |  | YP_004151935.1 | 33 | **0.01** |
| Theam_1334 | 2-C-methyl-D-erythritol 4-phosphate cytidylyltransferase | *ispD* | YP_004151938.1 | 26 | **0.08** |
| Theam_1337 | carbon-monoxide dehydrogenase, catalytic subunit | *codh* | YP_004151941.1 | 70 | **0.41** |
| Theam_1338 | Dinitrogenase iron-molybdenum cofactor biosynthesis protein | *nif* | YP_004151942.1 | 14 | **0.09** |
| Theam_1342 | Phosphoglycerate mutase | *pgm* | YP_004151946.1 | 24 | **0.09** |
| Theam_1343 | glutamate synthase (NADPH), homotetrameric | *gltA* | YP_004151947.1 | 53 | **0.56** |
| Theam_1344 | Protein of unknown function DUF2168 |  | YP_004151948.1 | 21 | **0.06** |
| Theam_1351 | hypothetical protein |  | YP_004151955.1 | 20 | **0.01** |
| Theam_1352 | fimbrial protein pilin |  | YP_004151956.1 | 20 | **0.33** |
| Theam_1354 | RNA-metabolising metallo-beta-lactamase |  | YP_004151958.1 | 52 | **0.01** |
| Theam_1356 | leucyl-tRNA synthetase | *leuS* | YP_004151960.1 | 105 | **0.19** |
| Theam_1357 | hypothetical protein |  | YP_004151961.1 | 20 | **0.06** |
| Theam_1359 | selenium metabolism protein YedF |  | YP_004151963.1 | 22 | **0.02** |
| Theam_1363 | Tetratricopeptide TPR_1 repeat-containing protein |  | YP_004151967.1 | 30 | **0.01** |
| Theam_1364 | GTP cyclohydrolase I | *folE* | YP_004151968.1 | 21 | **0.49** |
| Theam_1366 | hypoxanthine phosphoribosyltransferase | *hgprt* | YP_004151970.1 | 19 | **0.02** |
| Theam_1368 | argininosuccinate synthase | *argG* | YP_004151972.1 | 45 | **0.36** |
| Theam_1369 | trigger factor | *tig* | YP_004151973.1 | 49 | **0.13** |
| Theam_1372 | phosphoribosylformylglycinamidine synthase II | *fgams* | YP_004151976.1 | 82 | **0.24** |
| Theam_1373 | Domain of unknown function DUF1931 |  | YP_004151977.1 | 17 | **0.37** |
| Theam_1375 | Protein of unknown function DUF2148 |  | YP_004151979.1 | 19 | **0.13** |
| Theam_1378 | 2-oxoglutarate ferredoxin oxidoreductase subunit alpha | *oorA1* | YP_004151982.1 | 60 | **0.03** |
| Theam_1379 | 2-oxoglutarate ferredoxin oxidoreductase subunit beta | *oorB1* | YP_004151983.1 | 30 | **0.02** |
| Theam_1385 | flagellar hook-associated protein FlgK | *flgH* | YP_004151989.1 | 49 | **0.01** |
| Theam_1387 | type IV pilus assembly PilZ |  | YP_004151991.1 | 26 | **0.04** |
| Theam_1390 | MglA protein |  | YP_004151994.1 | 22 | **0.15** |
| Theam_1391 | Roadblock/LC7 family protein |  | YP_004151995.1 | 18 | **0.08** |
| Theam_1392 | pyruvate synthetase / thiamine pyrophosphate TPP-binding domain-containing protein | *porB* | YP_004151996.1 | 36 | **0.43** |
| Theam_1393 | pyruvate synthetase flavodoxin/ferredoxin oxidoreductase domain protein | *porA* | YP_004151997.1 | 45 | **0.46** |
| Theam_1394 | pyruvate synthetase ferredoxin/flavodoxin oxidoreductase, delta subunit | *porD* | YP_004151998.1 | 11 | **0.16** |
| Theam_1395 | pyruvate synthetase /ketoisovalerate oxidoreductase, gamma subunit | *porG* | YP_004151999.1 | 21 | **0.51** |
| Theam_1400 | SurA domain |  | YP_004152004.1 | 49 | **0.00** |
| Theam_1401 | YicC-like domain-containing protein |  | YP_004152005.1 | 33 | **0.17** |
| Theam_1402 | guanylate kinase |  | YP_004152006.1 | 26 | **0.03** |
| Theam_1403 | DNA-directed RNA polymerase, omega subunit | *rpoZ* | YP_004152007.1 | 8 | **0.10** |
| Theam_1404 | Dihydroorotate dehydrogenase, electron transfer subunit, iron-sulphur cluster binding domain |  | YP_004152008.1 | 28 | **0.06** |
| Theam_1408 | succinyl-CoA synthetase, beta subunit | *sucD* | YP_004152012.1 | 42 | **0.58** |
| Theam_1409 | succinyl-CoA synthetase, alpha subunit | *sucC* | YP_004152013.1 | 32 | **0.80** |
| Theam_1411 | 2-oxoglutarate synthetase ferredoxin subunit alpha | *oorA* | YP_004152015.1 | 42 | **0.64** |
| Theam_1412 | 2-oxoglutarate synthetase ferredoxin subunit beta | *oorB* | YP_004152016.1 | 32 | **0.48** |
| Theam_1413 | 2-oxoglutarate synthetase ferredoxin gamma subunit | *oorC* | YP_004152017.1 | 22 | **0.47** |
| Theam_1418 | adenosylmethionine-8-amino-7-oxononanoate aminotransferase | *bioA* | YP_004152022.1 | 50 | **0.04** |
| Theam_1419 | hypothetical protein |  | YP_004152023.1 | 18 | **0.22** |
| Theam_1420 | UspA domain-containing protein |  | YP_004152024.1 | 17 | **0.12** |
| Theam_1421 | protein of unknown function DUF814 |  | YP_004152025.1 | 37 | **0.01** |
| Theam_1422 | ribosomal subunit interface protein | *yfiA* | YP_004152026.1 | 21 | **0.11** |
| Theam_1423 | protein of unknown function DUF820 |  | YP_004152027.1 | 21 | **0.04** |
| Theam_1424 | GTP-binding protein LepA | *lepA* | YP_004152028.1 | 67 | **0.01** |
| Theam_1428 | hypothetical protein |  | YP_004152031.1 | 151 | **0.03** |
| Theam_1429 | helicase domain protein |  | YP_004152032.1 | 123 | **0.01** |
| Theam_1430 | Inorganic diphosphatase | *ppa* | YP_004152033.1 | 20 | **0.22** |
| Theam_1435 | PHP domain protein |  | YP_004152038.1 | 24 | **0.03** |
| Theam_1446 | fagellar hook-basal body protein- hook structural protein | *flgE* | YP_004152049.1 | 57 | **0.07** |
| Theam_1470 | Citrate transporter |  | YP_004152072.1 | 49 | **0.00** |
| Theam_1472 | ribosome biogenesis GTP-binding protein YsxC |  | YP_004152074.1 | 22 | **0.02** |
| Theam_1473 | Redoxin domain protein |  | YP_004152075.1 | 22 | **0.10** |
| Theam_1474 | thioredoxin reductase | *trxr* | YP_004152076.1 | 33 | **0.13** |
| Theam_1475 | thioredoxin | *trx* | YP_004152077.1 | 12 | **0.39** |
| Theam_1476 | regulatory protein ArsR |  | YP_004152078.1 | 11 | **0.04** |
| Theam_1477 | protein of unknown function DUF52 |  | YP_004152079.1 | 29 | **0.11** |
| Theam_1479 | Myo-inositol-1-phosphate synthase |  | YP_004152081.1 | 40 | **0.20** |
| Theam_1480 | hypothetical protein |  | YP_004152082.1 | 15 | **0.01** |
| Theam_1481 | tol-pal system protein YbgF |  | YP_004152083.1 | 26 | **0.05** |
| Theam_1482 | 2-nitropropane dioxygenase NPD | *npd* | YP_004152084.1 | 39 | **0.01** |
| Theam_1484 | peptidase M16 domain protein |  | YP_004152086.1 | 45 | **0.02** |
| Theam_1486 | phosphoribosylformylglycinamidine cyclo-ligase | *purM* | YP_004152088.1 | 37 | **0.13** |
| Theam_1487 | metal dependent phophohydrolase | *hdig* | YP_004152089.1 | 37 | **0.00** |
| Theam_1493 | NADH dehydrogenase subunit I | *nuoI* | YP_004152095.1 | 22 | **0.03** |
| Theam_1495 | NADH dehydrogenase (quinone) | *nuoD* | YP_004152097.1 | 43 | **0.03** |
| Theam_1497 | NADH-quinone oxidoreductase, B subunit | *nuoB* | YP_004152099.1 | 18 | **0.04** |
| Theam_1499 | phosphate ABC transporter, ATPase subunit | *pstB* | YP_004152101.1 | 29 | **0.04** |
| Theam_1502 | DNA polymerase III, alpha subunit | *polc* | YP_004152104.1 | 131 | **0.00** |
| Theam_1503 | acetyl-CoA carboxylase, carboxyl transferase, alpha subunit | *accA* | YP_004152105.1 | 36 | **0.02** |
| Theam_1505 | DEAD/DEAH box helicase domain protein |  | YP_004152107.1 | 47 | **0.05** |
| Theam_1510 | D-3-phosphoglycerate dehydrogenase | *pgdh* | YP_004152112.1 | 59 | **0.40** |
| Theam_1512 | Holliday junction DNA helicase RuvA | *ruvA* | YP_004152114.1 | 21 | **0.02** |
| Theam_1517 | translation initiation factor, aIF-2BI family | *alF* | YP_004152119.1 | 39 | **0.12** |
| Theam_1518 | UspA domain-containing protein |  | YP_004152120.1 | 18 | **0.19** |
| Theam_1519 | MazG family protein | *mazG* | YP_004152121.1 | 31 | **0.03** |
| Theam_1520 | cysteinyl-tRNA synthetase | *cysS* | YP_004152122.1 | 55 | **0.09** |
| Theam_1526 | Nickel transport complex, NikM subunit, transmembrane |  | YP_004152128.1 | 29 | **0.12** |
| Theam_1527 | Carbohydrate-selective porin OprB |  | YP_004152129.1 | 47 | **0.07** |
| Theam_1539 | pyruvate carboxylase, biotin carboxylase | *pycA* | YP_004152140.1 | 53 | **0.09** |
| Theam_1540 | pyruvate carboxylase alpha subunit | *pycB* | YP_004152141.1 | 69 | **0.28** |
| Theam_1544 | chorismate synthase | *aroC* | YP_004152145.1 | 42 | **0.04** |
| Theam_1545 | ribosomal protein L19 | *rplS* | YP_004152146.1 | 14 | **0.89** |
| Theam_1548 | translation initiation factor IF-2 |  | YP_004152148.1 | 99 | **0.07** |
| Theam_1550 | 1-phosphofructokinase |  | YP_004152150.1 | 34 | **0.04** |
| Theam_1552 | ribonucleotide reductase |  | YP_004152152.1 | 41 | **0.16** |
| Theam_1554 | ribonucleoside-diphosphate reductase, alpha subunit |  | YP_004152154.1 | 87 | **0.17** |
| Theam_1555 | Redoxin domain protein |  | YP_004152155.1 | 18 | **0.11** |
| Theam_1559 | beta-lactamase domain protein |  | YP_004152159.1 | 29 | **0.01** |
| Theam_1560 | chemotaxis sensory transducer |  | YP_004152160.1 | 28 | **0.01** |
| Theam_1561 | 5-methyltetrahydropteroyltriglutamate--homocysteine S-methyltransferase | *metE* | YP_004152161.1 | 82 | **0.69** |
| Theam_1563 | pseudogene |  |  | 79 | **0.01** |
| Theam_1564 | ErfK/YbiS/YcfS/YnhG family protein |  | YP_004152163.1 | 41 | **0.01** |
| Theam_1566 | thioesterase superfamily protein |  | YP_004152165.1 | 21 | **0.67** |
| Theam_1567 | carbamoyl-phosphate synthase, large subunit | *cpsL* | YP_004152166.1 | 119 | **0.33** |
| Theam_1568 | Adenylate kinase |  | YP_004152167.1 | 19 | **0.01** |
| Theam_1569 | sun protein | *rsmB* | YP_004152168.1 | 50 | **0.02** |
| Theam_1570 | Metal-dependent hydrolase HDOD |  | YP_004152169.1 | 24 | **0.04** |
| Theam_1571 | Metal-dependent hydrolase HDOD |  | YP_004152170.1 | 25 | **0.06** |
| Theam_1576 | Carbonate dehydratase |  | YP_004152175.1 | 28 | **0.08** |
| Theam_1578 | DNA-directed RNA polymerase, beta' subunit | *rpoB1* | YP_004152177.1 | 167 | **0.34** |
| Theam_1579 | DNA-directed RNA polymerase, beta subunit | *rpoB* | YP_004152178.1 | 163 | **0.38** |
| Theam_1580 | ribosomal protein L7/L12 | *L12* | YP_004152179.1 | 13 | **0.38** |
| Theam_1581 | ribosomal protein L10 |  | YP_004152180.1 | 20 | **0.47** |
| Theam_1582 | ribosomal protein L1 | *rplA* | YP_004152181.1 | 26 | **0.37** |
| Theam_1583 | ribosomal protein L11 | *L11* | YP_004152182.1 | 15 | **0.11** |
| Theam_1584 | transcription termination/antitermination factor NusG | *nusG* | YP_004152183.1 | 28 | **0.04** |
| Theam_1587 | translation elongation factor Tu |  | YP_004152186.1 | 44 | **2.19** |
| Theam_1588 | hypothetical protein |  | YP_004152187.1 | 52 | **0.83** |
| Theam_1590 | 16S rRNA processing protein RimM |  | YP_004152188.1 | 22 | **0.01** |
| Theam_1591 | tRNA (guanine-N1)-methyltransferase | *trmD* | YP_004152189.1 | 29 | **0.02** |
| Theam_1596 | chorismate mutase |  | YP_004152194.1 | 40 | **0.02** |
| Theam_1598 | Imidazoleglycerol-phosphate dehydratase |  | YP_004152196.1 | 22 | **0.03** |
| Theam_1600 | glutamate-1-semialdehyde-2,1-aminomutase | *hemL* | YP_004152198.1 | 47 | **0.21** |
| Theam_1605 | ATP synthase F0, A subunit | *atpa* | YP_004152203.1 | 26 | **0.01** |
| Theam_1611 | OmpA/MotB domain protein |  | YP_004152209.1 | 26 | **0.01** |
| Theam_1612 | gamma-glutamyl phosphate reductase | *proA* | YP_004152210.1 | 47 | **0.22** |
| Theam_1626 | hypothetical protein |  | YP_004152223.1 | 34 | **0.02** |
| Theam_1627 | pyruvate:water dikinase | *pvtk* | YP_004152224.1 | 100 | **0.85** |
| Theam_1628 | glycyl-tRNA synthetase, beta subunit | *glyS* | YP_004152225.1 | 77 | **0.11** |
| Theam_1629 | glycyl-tRNA synthetase, alpha subunit | *glyQ* | YP_004152226.1 | 33 | **0.11** |
| Theam_1632 | exsB protein | *exsB* | YP_004152229.1 | 26 | **0.03** |
| Theam_1645 | hypothetical protein |  | YP_004152242.1 | 17 | **0.06** |
| Theam_1647 | glutamyl-tRNA(Gln) amidotransferase, A subunit | *gatA* | YP_004152244.1 | 53 | **0.41** |
| Theam_1648 | glutamyl-tRNA(Gln) amidotransferase, C subunit | *gatC* | YP_004152245.1 | 11 | **0.02** |
| Theam_1656 | H+transporting two-sector ATPase B/B' subunit | *atpB'* | YP_004152253.1 | 17 | **0.14** |
| Theam_1657 | ATP synthase F0, B subunit | *atpB* | YP_004152254.1 | 18 | **0.08** |
| Theam_1658 | ATP synthase F1, delta subunit | *atpD* | YP_004152255.1 | 20 | **0.05** |
| Theam_1659 | ATP synthase F1, alpha subunit | *atpA* | YP_004152256.1 | 55 | **0.16** |
| Theam_1660 | ATP synthase F1, gamma subunit | *atpC* | YP_004152257.1 | 32 | **0.10** |
| Theam_1661 | ATP synthase F1, beta subunit | *atpD* | YP_004152258.1 | 54 | **0.19** |
| Theam_1662 | ATP synthase F1, epsilon subunit | *atpE* | YP_004152259.1 | 16 | **0.12** |
| Theam_1667 | protein of unknown function DUF62 |  | YP_004152264.1 | 29 | **0.01** |
| Theam_1668 | glucose inhibited division protein A | *gidA* | YP_004152265.1 | 72 | **0.06** |
| Theam_1669 | methyltransferase GidB | *gidB* | YP_004152266.1 | 24 | **0.02** |
| Theam_1672 | homoserine kinase | *thrB* | YP_004152269.1 | 33 | **0.16** |
| Theam_1676 | valyl-tRNA synthetase | *valS* | YP_004152273.1 | 102 | **0.19** |
| Theam_1677 | CMP/dCMP deaminase zinc-binding |  | YP_004152274.1 | 17 | **0.01** |
| Theam_1678 | recA protein | *recA* | YP_004152275.1 | 38 | **0.02** |
| Theam_1679 | twitching motility protein | *pilT* | YP_004152276.1 | 41 | **0.03** |
| Theam_1682 | alanyl-tRNA synthetase | *alaS* | YP_004152279.1 | 99 | **0.18** |
| Theam_1684 | hypothetical protein |  | YP_004152281.1 | 150 | **0.02** |
| Theam_1685 | hypothetical protein |  | YP_004152282.1 | 50 | **0.00** |
| Theam_1686 | PfkB domain protein |  | YP_004152283.1 | 36 | **0.02** |
| Theam_1688 | transcription termination factor NusA | *NusA* | YP_004152285.1 | 43 | **0.08** |
| Theam_1689 | protein of unknown function DUF150 |  | YP_004152286.1 | 18 | **0.04** |
| Theam_1691 | RNA binding S1 domain protein |  | YP_004152288.1 | 39 | **0.01** |
| Theam_1696 | S-adenosyl-methyltransferase MraW | *mraW* | YP_004152293.1 | 34 | **0.02** |
| Theam_1697 | ornithine carbamoyltransferase | *otc* | YP_004152294.1 | 35 | **0.14** |
| Theam_1712 | stationary-phase survival protein SurE | *surE* | YP_004152309.1 | 28 | **0.03** |
| Theam_1725 | Indole-3-glycerol-phosphate synthase |  | YP_004152322.1 | 29 | **0.08** |
| Theam_1726 | UDP-N-acetylglucosamine pyrophosphorylase | *glmU* | YP_004152323.1 | 51 | **0.10** |
| Theam_1727 | NUDIX hydrolase |  | YP_004152324.1 | 17 | **0.07** |
| Theam_1728 | hypothetical protein |  | YP_004152325.1 | 32 | **0.01** |
| Theam_1729 | S-adenosylmethionine/tRNA-ribosyltransferase-isomerase | *queA* | YP_004152326.1 | 39 | **0.03** |
| Theam_1735 | hypothetical protein |  | ADU97691.1 | 43 | **0.03** |
| Theam_1738 | hypothetical protein |  | ADU97694.1 | 19 | **0.06** |
| Theam_1739 | hypothetical protein |  | ADU97695.1 | 23 | **0.03** |
| Theam_1742 | hypothetical protein |  | ADU97698.1 | 35 | **0.01** |
| Theam_1744 | hypothetical protein |  | ADU97700.1 | 22 | **0.01** |
| Theam_1768 | hypothetical protein |  | ADU97724.1 | 30 | **0.01** |
| Theam_1771 | ATPase associated with various cellular activities AAA_5 |  | ADU97727.1 | 31 | **0.04** |
| Theam_1774 | trichohyalin |  | ADU97730.1 | 21 | **0.05** |
| Theam_1778 | hypothetical protein |  | ADU97734.1 | 12 | **0.12** |
| Theam_1790 | hypothetical protein |  | ADU97746.1 | 63 | **0.01** |
| Theam_1795 | peptidase C11 clostripain |  | ADU97751.1 | 76 | **0.01** |
| Theam_1797 | hypothetical protein |  | ADU97753.1 | 45 | **0.06** |
| Theam_1799 | type II secretion system protein E | *gspE* | ADU97755.1 | 46 | **0.22** |
| Theam_1801 | hypothetical protein |  | ADU97757.1 | 19 | **0.05** |
| Theam_1803 | hypothetical protein |  | ADU97759.1 | 19 | **0.07** |
| Theam_1804 | type II secretion system protein E | *gspE* | ADU97760.1 | 60 | **0.01** |
| Theam_1805 | hypothetical protein |  | ADU97761.1 | 28 | **0.01** |
| Theam_1818 | hypothetical protein |  | ADU97774.1 | 18 | **0.06** |

Accession numbers refer to the *T. ammonificans* proteins in NCBI. NSAF% are normalized spectral abundance factors, i.e. relative abundances for each protein in % of all proteins in the sample. NSAF% are average values (n=3).
