# Supplementary material for: Insight into the evolution of microbial metabolism from the deep-branching bacterium, Thermovibrio ammonificans
Source: eLife. 2017 Apr 24;6:e18990. doi: 10.7554/eLife.18990 (PMC5441870; doi:10.7554/eLife.18990)
Supplement: Figure 9—source data 1. — DOI: http://dx.doi.org/10.7554/eLife.18990.019 [file elife-18990-fig9-data1.docx]

**Figure 5-source data 1.** Accession number of the ATP citrate lyase sequences used for the reconstruction of the phylogenetic history of the enzyme presented in Fig. 2 in the main text.

|  | **Accession numbers** | |
| --- | --- | --- |
| **Organism** | **AclB** | **AclA** |
| *Balnearium* *lithotrophicum* 17S | 114054980 | 114054981 |
| *Caminibacter* *mediatlanticus* TB2 | 494739975 | 494739977 |
| *Candidatus* Nitrospira defluvii | 302036204 | 302036205 |
| *Chlorobaculum* *parvum* NCIB 8327 | 193212663 | 193212664 |
| *Chlorobium* *chlorochromatii* CaD3 | 78188767 | 78188768 |
| *Chlorobium* limicola DSM 245 | 189346749 | 189346748 |
| *Chlorobium* *phaeobacteroides* DSM 266 | 119357118 | 119357117 |
| *Chlorobium* *phaeovibrioides* DSM 265 | 145219675 | 145219674 |
| *Chlorobium* *tepidum* TLS | 21673915 | 21673914 |
| *Chloroherpeton* *thalassium* ATCC 35110 | 193216250 | 193216249 |
| *Desulfurobacterium* sp. TC5-1 | 551219091 | 551219090 |
| *Desulfurobacterium* *thermolithotrophum* DSM 11699 | 325295317 | 325295316 |
| *Lebetimonas* sp. JS170 | 640061845 | 640076131 |
| *Nautilia* *profundicola* AmH | 224372752 | 224372753 |
| *Nitratifractor* *salsuginis* DSM 16511 | 319956330 | 319956331 |
| *Nitratiruptor* sp. SB155-2 | 152990401 | 152990402 |
| *Pelodictyon* *luteolum* DSM 273 | 78186923 | 78186924 |
| *Persephonella* *marina* EX-H1 | 225851245 | 225851244 |
| *Persephonella* sp. KM09-Lau-8 | 657725816 | 657725815 |
| *Prosthecochloris* *aestuarii* DSM 271 | 194333911 | 194333912 |
| *Sulfuricurvum* *kujiense* DSM 16994 | 313681794 | 313681795 |
| *Sulfurihydrogenibium* *azorense* Az-Fu1 | 225848355 | 225848356 |
| *Sulfurihydrogenibium* sp. YO3AOP1 | 188996978 | 188996977 |
| *Sulfurihydrogenibium* *subterraneum* DSM 15120 | 114055039 | 655805784 |
| *Sulfurimonas* *autotrophica* DSM 16294 | 307721497 | 307721496 |
| *Sulfurimonas* *denitrificans* DSM 1251 | 78776769 | 78776770 |
| *Sulfurimonas* *gotlandica* GD1 | 495611782 | 495610702 |
| *Sulfurovum* sp. AR | 495520539 | 495520532 |
| *Sulfurovum* sp. NBC37-1 | 152992137 | 152992138 |
| *Thermovibrio* *ammonificans* HB-1 | 319789997 | 319789996 |
| Uncultured *Sulfuricurvum* sp. RIFRC-1 | 476409849 | 476409850 |
| *Aquifex* *aeolicus* VF5^1^ | 15606514 | 15606916 + 15605724 |
| *Hydrogenobacter* *thermophilus* TK-6^1^ | 384129780 | 384128288 + 384128393 |

1- Citryl-CoA synthase subunit A (CcsA) was used in place of AclB and citryl-CoA synthase subunit B (CcsB) was manually concatenated to citryl-CoA lyase (Ccl) in place of AclA to reconstruct a hypothetical ancestral ATP citrate lyase enzyme.
